# Arterial aging and the autonomic nervous system: is the relationship differently modified by physical activity in men and women?

**DOI:** 10.3389/fragi.2025.1653656

**Published:** 2025-09-24

**Authors:** Alessandro Sestu, Alessandro Lai, Veronica Murru, Agnese Favale, Angelo Scuteri

**Affiliations:** ^1^ Department of Medical Sciences and Public Health – University of Cagliari, Cagliari, Italy; ^2^ Post Graduate Medical School of Internal Medicine – University of Cagliari, Cagliari, Italy; ^3^ Post Graduate Medical School of Geriatrics – University of Cagliari, Cagliari, Italy; ^4^ Internal Medicine Unit – University Hospital Monserrato - Azienda Ospedaliero-Universitaria di Cagliari, Cagliari, Italy

**Keywords:** arterial stiffness, autonomic nervous system, physical activity, pulse wave velocity, sex

## Abstract

**Introduction:**

Arterial aging is an independent risk factor for cardiovascular morbidity and mortality and is beneficially influenced by physical activity. However, it remains unclear whether the impact of physical activity on arterial stiffness differs between men and women and whether selected factors contribute to sex differences in the association of physical activity with arterial aging.

**Methods:**

Data from healthy volunteers (n = 265; mean age: 40 ± 16 years, 42.6% women) were used. Arterial aging was assessed using carotid-to-femoral pulse wave velocity (PWV). Volunteers were categorized as sedentary (no regular weekly physical activity) and regularly active.

**Results:**

Physically active men presented a significantly lower PWV than the sex-matched sedentary group (8.2 ± 0.2 versus 9.0 ± 0.3 m/s, *p* < 0.01). In the fully adjusted model (adjusted for age, blood pressure, heart rate, muscular mass, fat mass, and visceral adiposity), a steeper association between PWV and autonomic nervous system activity was observed in sedentary individuals than in physically active men. Physical activity was associated with no difference in PWV (7.9 ± 0.3 versus 7.9 ± 0.2 m/s), and no significant association between PWV and autonomic nervous system activity was observed in women.

**Conclusion:**

Physical activity was associated with a lower increase in arterial aging, indexed as pulse wave velocity, for any increase in autonomic nervous system activity in men. This effect was independent of age, blood pressure, and adiposity. The same effect was not observed in women. Future studies should clarify how these findings may inform a personalized approach to cardiovascular (CV) risk reduction.

## Introduction

Pulse wave velocity (PWV) is a reliable and reproducible index of large artery stiffness in clinical settings (Van Bortel et al., 2012). It has also emerged as a proxy of arterial aging, capturing the continuum from early (accelerated) vascular aging (EVA) (Nilsson et al., 2018) to healthy vascular aging (HVA), i.e., “slower than average” arterial aging (Nilsson et al., 2018). Greater PWV values are associated with functional limitations (Scuteri et al., 2021) and increased cardiovascular damage (Kario et al., 2020). Thus, PWV is now accepted as a proxy of arterial aging (Calimport et al., 2019).

Although multiple factors are implicated in increased large artery aging, the precise interplay and underlying mechanisms remain a complex and active area of investigation. Arterial stiffening is strongly associated with higher blood pressure levels (Van Bortel et al., 2012) and involves the interplay of blood pressure and structural (i.e., central arterial remodeling) changes in vascular wall (AlGhatrif et al., 2023) and content (M et al., 2024). Adiposity is associated with greater arterial aging (Brunner et al., 2015). The autonomic nervous system represents an additional contributor to arterial aging. Correlational data suggest a pressure-independent relationship between peripheral sympathetic outflow and central PWV in young healthy subjects (Van der Berg et al., 2016).

Together with these risk factors, physical exercise is considered beneficial for arterial aging: higher levels of physical activity are associated with lower PWV (Vandercappellen et al., 2020; Ahmadi-Abhari et al., 2017).

Factors underlying the association between physical activity and arterial aging are thought to include reductions in blood pressure (Hansen et al., 2022), adiposity (Ismail et al., 2012), and modulation of autonomic nervous system activity (Ino-Oka et al., 2009; Swierblewska et al., 2010).

We hypothesized that there are sex differences in the association between physical activity and arterial aging and that a more precise characterization of factors underlying these sex differences may contribute to a personalized approach to the prevention of CV events in men and women.

Thus, the aims of the present study were to identify i) sex differences in the association between physical activity and arterial aging and ii) factors contributing to the above-mentioned differences in men and women.

## Methods

The data that support the findings of this study are available from the corresponding author upon reasonable request for researchers who meet the criteria for access to confidential data.

The study complies with the Declaration of Helsinki, and the research protocol was approved by the locally appointed ethics committee (Prot. NP/2022/4588).

### Subjects

The study population consisted of healthy volunteers. Subjects were excluded if they had cancer, acute myocardial infarction in the previous 6 months, clinical overt heart failure, liver failure (AST and ALT >50 IU/mL and/or serum bilirubin >3 mg/dL), serum creatinine ≥ 2 mg/dL, secondary hypertension, or thyroid disease (hypo- or hyperthyroidism requiring hormonal treatment). Additional exclusion criteria included a history of large-vessel stroke and atrial fibrillation since their presence interferes with the accuracy of aorta stiffness measurements. After informed consent was provided, all patients underwent a medical history, a clinic visit, and measurements of body composition, heart rate variability (HRV), and arterial aging.

### Physical activity

A self-administered questionnaire was also used to collect information on physical activity. The first question in the questionnaire exploring physical activity was “How many days a week do you perform a moderate to vigorous physical activity such as walking at a fast speed?”; the second question exploring physical activity was “On average, how many minutes per week do you dedicate to these activities?”. Every participant provided a clear response to question 1 (how many days per week). However, approximately one-third of the participants in the present study were not able to provide a clear and detailed answer to the second question. Therefore, participants were classified as follows: sedentary, if they reported no physical activity during the week; physically active, if they reported regular physical activity at least once a week.

The adopted definition differs from that adopted by the World Health Organization (WHO), which defines physical activity as “at least 150 min of moderate-intensity physical activity per week” (https://www.who.int/news-room/fact-sheets/detail/physical-activity). Implications are discussed in the limitations of the study.

### Anthropometric and body composition assessment

Height, weight, and waist circumference were determined for all participants. Body mass index (BMI) was calculated as body weight (kg)/height (m^2^).

A bioelectrical impedance measure was performed using the Tanita MC-780^®^ (Tanita Europe BV, Amsterdam, Netherlands) multi-frequency segmental body composition analyzer under standardized conditions: ambient temperature between 23 °C and 25 °C, fasting for 3 h, and an empty bladder. The device acquired measurements of total fat mass, body fat percentage, total muscle mass, skeletal muscle mass, and visceral fat, along with total, intracellular, and extracellular water.

### Blood pressure measurement

Blood pressure was measured in the non-dominant arm with a sphygmomanometer using an appropriately sized cuff after a 10-min rest in a dark, quiet room. The average of the second and third measurements for systolic blood pressure (SBP) and diastolic blood pressure (DBP) was used in the analysis. Pulse pressure was computed as PP= (SBP−DBP); mean BP was computed as mean blood pressure (MBP) = DBP +(PP/3).

### Assessment of pulse wave velocity

Arterial aging was assessed non-invasively using the carotid–femoral PWV.

PWV was automatically determined as foot-to-foot velocity using the validated SphygmoCor^®^ device (AtCor Medical, Cardiex, Australia). The pulse transit time was determined as the average of 10 consecutive beats. The distance travelled by the pulse wave was measured over the body surface as the distance between the two recording sites (carotid and femoral pulse). The carotid location–sternal notch distance was subtracted from the carotid–femoral site distance. PWV was calculated as the ratio of distance to transit time.

To account for the known influence of distending blood pressure on PWV, PWV was also normalized for MBP (PWV/MBP).

### Heart rate variability

HRV was measured using AtCor Medical HRV software to assess sympathetic/parasympathetic autonomic function. A standard 2-min electrocardiogram recording was performed with the patient in the supine position, maintaining a regular and calm breathing pattern (5-min resting period) in a quiet room.

Spectral analysis of a series of consecutive RR intervals allowed the quantification of sympathetic and vagal influences on the heart. The evaluated parameters were as follows:

– power spectral density at the high-frequency (HF) range (0.15–0.4 Hz);
– power spectral density at the low-frequency (LF) range (0.04–0.15 Hz).

Vagal activity is the major contributor to the HF component and time-domain parameters, while LF reflects both sympathetic and vagal activities (Ismail et al., 2012).

### Statistical analysis

All analyses were performed using SAS University Edition.

Univariate regression analysis with Pearson’s r was calculated using PROC CORR.

ANCOVA was used to calculate age-adjusted least-square means of study variables. Therefore, data are presented as least-square means ± standard error (SEM), with *post hoc* Bonferroni group comparisons. PROC ANCOVA was also used to evaluate the interaction between sex and physical activity.

Multivariable models were constructed to identify factors associated with PWV. These models included the following parameters as covariates: age, blood pressure, heart rate, muscular and fat mass, visceral adiposity, LF, and HF.

An analysis of covariance was used to test whether the slope of the association between PWV and autonomic nervous system activity differed in younger (≤50 years) or older (>50 years) subjects. Interaction terms were constructed between autonomic nervous system activity and age group, sex, and physical activity. A significant triple interaction term indicated that the effect of sex and physical activity on arterial stiffness differed in young and older subjects.

A two-sided *p*-value <0.05 indicated statistical significance.

## Results

### Physical activity differentially impacts arterial aging, blood pressure, adiposity, and the autonomic nervous system in men and women

The study population consisted of 265 volunteers: 152 men with a mean age of 39.3 ± 17.8 years and 113 women with a mean age of 40 ± 16 years.

To better identify possible sex differences in the impact of physical activity on arterial aging and its possible determinants, participants were divided into four subgroups depending on sex and physical activity. Age significantly differed in the four groups; therefore, age-adjusted means were calculated and are illustrated in Table 1.

**TABLE 1:** Comparison of study variables in men and women stratified by physical activity status (age-adjusted least-squares means ± SEM).

	Men		Women		Within sex		*Between sex*	
	Sedentary (45)	Physically active (107)	Sedentary (40)	Physically active (73)	Men	Women	*Not active*	*Active*
	(1)	(2)	(3)	(4)	1 vs. 2	3 vs. 4	*1* vs*. 3*	*2* vs*. 4*
Age (years)	49.8 ± 18.1	34.9 ± 15.8	39.8 ± 15.9	40.4 ± 16.1	—			
PWV (m/sec)	9.0 ± 0.3	8.2 ± 0.2	7.9 ± 0.3	7.9 ± 0.2	**0.01**	**0.90**	*0.0028*	*0.17*
PWV/MBP (m/sec*mmHg)	9.4 ± 0.3	8.9 ± 0.2	8.5 ± 0.3	8.6 ± 0.2	**0.11**	**0.67**	*0.024*	*0.42*
SBP (mmHg)	130.5 ± 2.0	128.8 ± 1.2	119.0 ± 1.9	119.0 ± 1.4	**0.47**	**0.98**	*0.0001*	*0.0001*
DBP (mmHg)	78.7 ± 1.4	75.5 ± 0.8	79.7 ± 1.3	77.9 ± 1.0	**0.05**	**0.28**	*0.61*	*0.06*
HR (bpm)	76.3 ± 1.8	68.1 ± 1.1	73.8 ± 1.7	71.8 ± 1.4	**0.0002**	**0.049**	*0.32*	*0.035*
BMI (Kg/m^2^)	25.3 ± 0.6	24.1 ± 0.3	22.4 ± 0.5	23.3 ± 0.4	**0.07**	**0.19**	*0.0003*	*0.13*
Waist circumference (cm)	95.2 ± 1.5	88.4 ± 0.9	82.1 ± 1.4	81.9 ± 1.0	**0.0001**	**0.91**	*0.0001*	*0.0001*
Fat mass (Kg)	18.2 ± 1.0	13.2 ± 0.6	16.1 ± 0.9	15.9 ± 0.7	**0.0001**	**0.85**	*0,14*	*0.03*
Muscle mass (Kg)	55.6 ± 1.1	57.7 ± 0.6	40.8 ± 1.0	42.8 ± 0.7	**0.09**	**0.10**	*0.0001*	*0.0001*
Visceral fat	8.0 ± 0.4	5.6 ± 0.2	3.6 ± 0.4	3.6 ± 0.3	**0.0001**	**0.98**	*0.0001*	*0.0001*
Total body water (%)	55.5 ± 0.7	57.8 ± 0.4	49.5 ± 0.6	49.9 ± 0.4	**0.0023**	**0.69**	*0.0001*	*0.0001*
Phase angle (°)	6.1 ± 0.1	6.4 ± 0.1	5.2 ± 0.1	5.5 ± 0.1	**0.0036**	**0.0001**	*0.0001*	*0.0001*
Sarcopenic index (Kg/m^2^)	8.1 ± 0.2	8.4 ± 0.1	6.3 ± 0.2	6.8 ± 0.1	**0.12**	**0.0001**	*0.0001*	*0.0001*
LF	1166 ± 124	1109 ± 71	1070 ± 111	1123 ± 82	**0.69**	**0.77**	*0.57*	*0.90*
HF	361 ± 42	358 ± 24	274 ± 37	324 ± 28	**0.95**	**0.28**	*0.12*	*0.35*

PWV, pulse wave velocity; PWV/MBP, pulse wave velocity normalized for mean blood pressure; SBP, systolic blood pressure; DBP, diastolic blood pressure; HR, heart rate; BMI, body mass index; LF, power spectral density at the low frequency; HF, power spectral density at the high frequency.

Physically active men presented a significantly lower PWV than the sex-matched sedentary group (8.2 ± 0.2 versus 9.0 ± 0.3 m/s, *p* < 0.01), with no differences in blood pressure. Waist circumference, visceral adiposity, and HR were also lower in physically active men than in sedentary men. When compared to sedentary women, physical activity was not associated with differences in PWV. A lower HR was observed in physically active women than in sedentary controls. Both physically active and sedentary men showed higher SBP, waist circumference, and visceral adiposity than paired women.

### Sex differences in the determinants of arterial aging

Univariate regression analyses showed that determinants of PWV differed in men and women. As illustrated in Table 2, blood pressure and adiposity were significantly associated with PWV in both men and women. Conversely, physical activity was negatively associated with PWV in men (the greater the physical activity, the lower the PWV) but did not appear as a significant determinant in women. HR, LF, and HF were significantly and positively associated with PWV in men, but not in women.

**TABLE 2:** Univariate regression analyses correlating PWV with physical activity, age, blood pressure, heart rate, body composition, and autonomic nervous system activity.

	Men		Women	
	*r*	*p*	r	p
Age (years)	*0.676*	*0.0001*	0.595	0.0001
Sedentary (yes vs. no)	*0.392*	*0.0001*	−0.024	0.804
Daily physical activity	*−0.350*	*0.0001*	0.031	0.789
SBP (mmHg)	*0.396*	*0.0001*	0.453	0.0001
DBP (mmHg)	*0.499*	*0.0001*	0.380	0.0001
HR (bpm)	*0.230*	*0.008*	0.008	0.947
BMI (Kg/m^2^)	*0.306*	*0.0001*	0.288	0.002
Waist circumference (cm)	*0.487*	*0.0001*	0.389	0.0001
Fat mass	*0.370*	*0.0001*	0.301	0.001
Muscle mass *(Kg)*	*−0.154*	*0.053*	0.095	0.319
Visceral fat *(Kg)*	*0.581*	*0.0001*	0.492	0.0001
Total body water (%)	*−0.093*	*0.257*	0.069	0.467
Phase angle *(°)*	*−0.470*	*0.0001*	−0.122	0.171
Sarcopenic index (Kg/m^2^)	*−0.115*	*0.174*	0.131	0.201
LF	*0.177*	*0.030*	0.125	0.188
HF	*0.189*	*0.020*	0.107	0.260

PWV, pulse wave velocity; PWV/MBP, pulse wave velocity normalized for mean blood pressure; SBP, systolic blood pressure; DBP, diastolic blood pressure; HR, heart rate; BMI, body mass index; LF, power spectral density at the low frequency; HF, power spectral density at the high frequency.

### Physical activity impacts on the association of arterial aging with autonomic activity, but not with blood pressure or adiposity, in a sex-specific manner

To investigate potential sex differences in the association between arterial aging and its possible determinants, multivariable regression models were constructed. They revealed no significant differences in the association between age, blood pressure, fat mass, or visceral adiposity and PWV between men and women, sedentary or physically active (Supplementary Figure S1).

**FIGURE 1:**
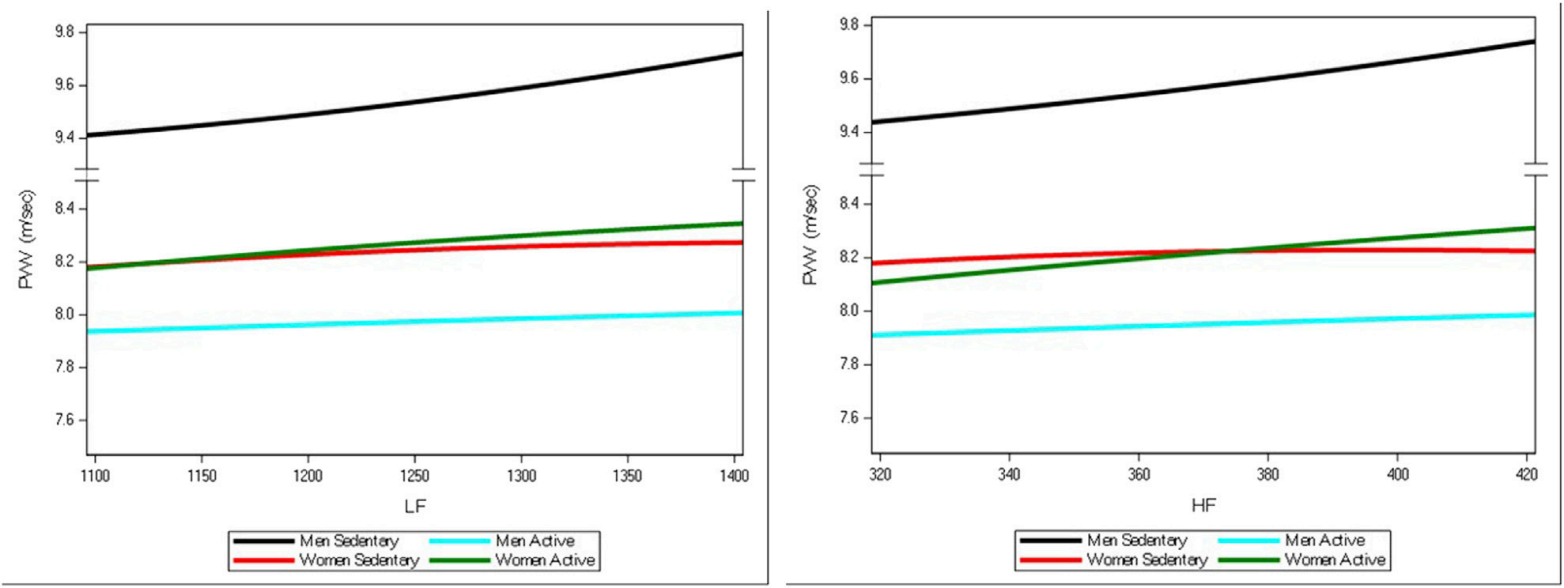
Smoothed lines for the association between PWV and autonomic nervous activity, indexed by LF or HF, in sedentary or physically active men and women—after controlling for age, blood pressure, HR, and adiposity. Black lines represent sedentary men. Blue lines represent physically active men. Red lines represent sedentary women. Green lines represent physically active women.

LF (t = 2.67, p = 0.081) and HF (t = 2.51, p = 0.0125) were significantly associated with PWV in the multivariable regression models. However, their association with PWV differed in the four groups (*p* for interaction = 0.017 for LF and *p* = 0.027 for HF). As illustrated in Figure 1, the slopes of the association between PWV and autonomic activity, indexed by LF or HF, differed in sedentary or physically active men and women—after controlling for age, blood pressure, HR, and adiposity. In more detail, sedentary men showed a steeper increase in PWV with any increase in LF or HF than physically active men, which showed an association between PWV and LF or HF similar to that observable in women. No significant physical difference in the slope of the association between PWV and LF or HF was observed between active and sedentary women.

Similar findings were observed when waist or fat mass was—alternatively—added or substituted for visceral adiposity in the multivariable regression models.

We also sought to explore whether menopause could impact the abovementioned sex differences. Therefore, we first divided subjects into two age groups: ≤50 and >50 years. Then, an interaction term between age group, sex, physical activity, and the autonomic nervous system was introduced into the multivariable regression models. The interaction term did not reach statistical significance, suggesting that the abovementioned association—as illustrated in Figure 1—did not differ between men and women younger or older than 50 years.

## Discussion

Physical inactivity is a common condition. Recently, the WHO estimated that more than 1 out of 4 adults is physically inactive, i.e., has a weekly physical activity of less than 150 min of moderate-intensity or 75 min of vigorous-intensity aerobic activity per week (Guthold et al., 2018; Bull et al., 2020). The consequences of physical inactivity on cardiometabolic diseases make this an important modifiable risk factor for the primary prevention of cardiometabolic diseases (Eijsvogels et al., 2016).

It is a common belief that the beneficial effects of physical activity are attributable to calorie consumption and the related weight loss. However, cardioprotective effects of exercise are mediated by multiple and complex mechanisms, largely independent of calorie consumption. Physical activity reduces inflammation (Goldhammer et al., 2005). Additionally, physical activity promotes metabolic flexibility, i.e., the ability to efficiently switch between using carbohydrates and fats as fuel sources, which is important for overall metabolic health and energy balance (Brun et al., 2022).

If the benefits of physical activity largely do not rely on calorie reduction and weight loss, it is expected that, when compared to sedentary peers, physically active subjects may not show a significant difference in average body weight.

The results of the present study should be read by analogy with the abovementioned conceptual frame. Compared to sedentary peers, physically active men and women did not show significant differences in the average autonomic nervous system activity, along with blood pressure or BMI.

However, in men, physical activity modified the association between autonomic nervous system activity and arterial aging, indexed as PWV. In other terms, for a given level of autonomic nervous system activity—indexed by LF or HF—physically active men showed significantly lower arterial aging than sedentary men. No difference was observed between sedentary and active women. This association was independent of age, blood pressure, HR, and adiposity.

In other terms, it is like that physical activity modifies the “set point” of the relationship between autonomic nervous system activity and arterial stiffness only in men, but not in average LF and HF activity, which was similar in men and women, sedentary or active.

The benefits of physical activity on arterial aging were reported. Arterial aging is positively associated with sedentary behavior and inversely associated with physical activity in cross-sectional studies (Van der Berg et al., 2016; Vaitkevicius et al., 1993; Parsons et al., 2016; Horta et al., 2015; Garcia-Hermoso et al., 2015). Greater physical activity was associated with a smaller 5-year increase in PWV, and a greater time spent sitting was associated with faster PWV progression in the Whitehall II cohort (Vandercappellen et al., 2020), independent of conventional vascular risk factor levels.

Mechanisms underlying the benefits of physical activity on arterial aging are thought to involve BP reduction, adiposity, and the autonomic nervous system.

Physical activity is accompanied by an average SBP reduction of 2–4 and 5–8 mmHg in subjects with normotension and hypertension, respectively (Hansen et al., 2022). Notably, the reduction in SBP occurs irrespective of age (Williamson et al., 2016) or sex (Igarashi et al., 2018). Blood pressure levels also impact structural vascular changes known to be associated with arterial aging (M et al., 2024). The benefits of aerobic exercise on adiposity reduction were established (Ismail et al., 2012; Steeves et al., 2012). Physical activity may influence adipose tissue depots differently in men and women (Smith et al., 2013). It has been observed that physical activity reduces subcutaneous and visceral adipose tissue volumes. This association was observed in both men and women, but the effect was significantly greater in women than in men (Murabito et al., 2015). Physical activity may modulate autonomic nervous system activity and, thus, result in less arterial aging (Ino-Oka et al., 2009; Swierblewska et al., 2010).

Our study showed sex differences in the effect of physical activity on the association between autonomic nervous system activity and arterial aging, which was independent of age, blood pressure, heart rate, and adiposity. This novel observation expands our knowledge of sex differences in the autonomic regulation of the cardiovascular system.

A steeper age-related increase in sympathetic activity in men than in women has been reported (Matsukawa et al., 2020; Sugiyama et al., 1998). These changes were not associated with an increase in the resting heart rate (Matsukawa et al., 2020). We have previously reported that the relationship between lower autonomic nervous system activity and arterial aging was significant in women but not in men (Serra et al., 2022). Additionally, the magnitude of the association was greater in women with than in those without diabetes; diabetes did not impact the association between the autonomic nervous system and arterial aging (Serra et al., 2022). In the presence of chronic kidney disease, a significant inverse association between sympathetic activity and arterial aging was observed in women. No association between sympathetic activity and PWV was reported in men, who had higher baseline sympathetic activity than female subjects (Zanuzzi et al., 2024).

The major strength of the present study is that healthy volunteers are the study population. However, the present study has several limitations. The first limitation is represented by its cross-sectional design, which does not allow the identification of factors underlying the reported sex difference in the effect of physical activity on the association between the autonomic nervous system and arterial aging. An additional limitation is the use of a physical activity measurement definition that differs from the WHO recommendation. This may impact the comparability of our study. The duration of electrocardiographic recording time for HRV measurement is relatively short. It has been reported that the HRV frequency domain is typically recorded over a minimum 2-min period (Task Force Report, 1996). This ease of recording has led to short-term HRV measurements being the most commonly reported source of published HRV data (Shaffer et al., 2014). Notably, our observed values are within the normative data from short-term HRV studies (Nunan et al., 2010). The values of HRV at 24 h, short-term, and ultra-short-term durations are not interchangeable. Finally, the size of the study population did not allow a more comprehensive analysis of the potential effects of menopause on sex differences in the observed association between the autonomic nervous system and arterial stiffness.

### Perspectives

The present study showed that more physical activity was associated with lower arterial aging, indexed as carotid–femoral PWV, for any given autonomic nervous system activity in physically active men compared to sedentary men. However, this association did not differ between sedentary and physically active women.

Future research should aim to understand the biological pathways underlying the effect of physical activity on the negative association between autonomic nervous system activity and arterial aging in men and women. This new knowledge may eventually contribute to a more personalized approach to the prevention of CV events and a better understanding of vascular aging.

## Data Availability

The data that support the findings of this study are available from the corresponding author upon reasonable request for researchers who meet the criteria for access to confidential data. Requests to access the datasets should be directed to ANGELOELEFANTE@INTERFREE.IT.
