# Supplementary figures and images for: Arterial aging and the autonomic nervous system: is the relationship differently modified by physical activity in men and women?
Source: Front Aging. 2025 Sep 24;6:1653656. doi: 10.3389/fragi.2025.1653656 (PMC12504498; doi:10.3389/fragi.2025.1653656)

# Panel C

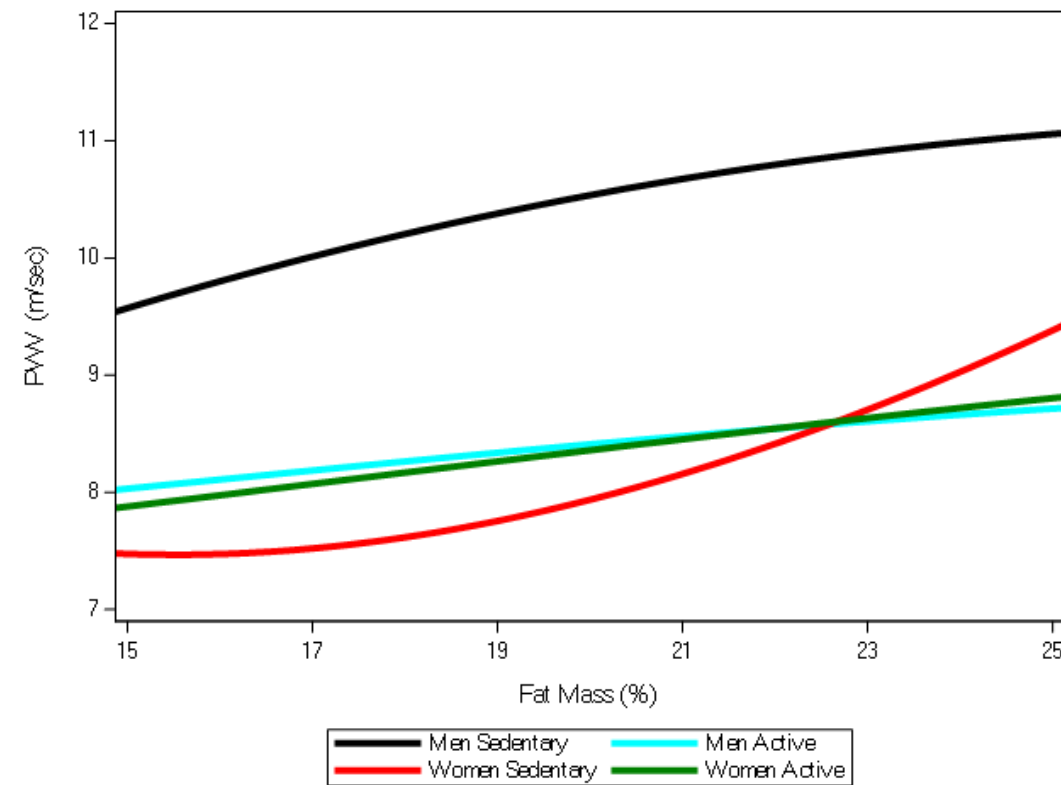

# Panel D

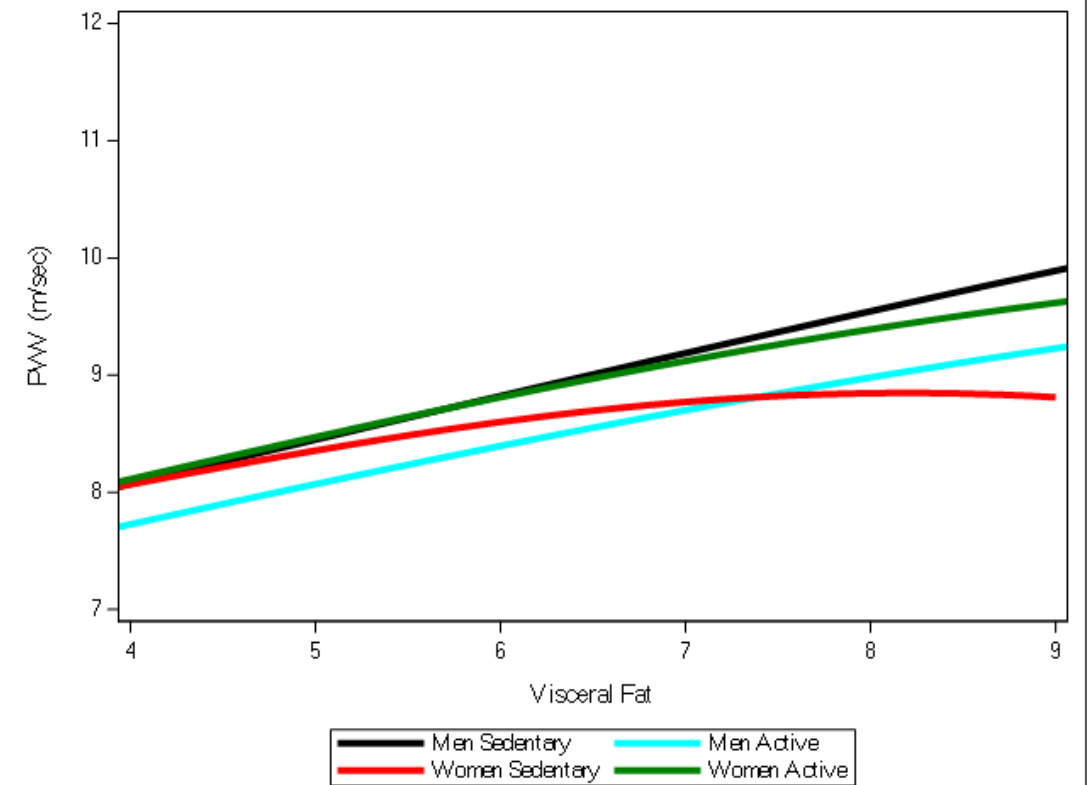

Supplement: Supplementary file 1 [file DataSheet2.pdf]

## Panel A

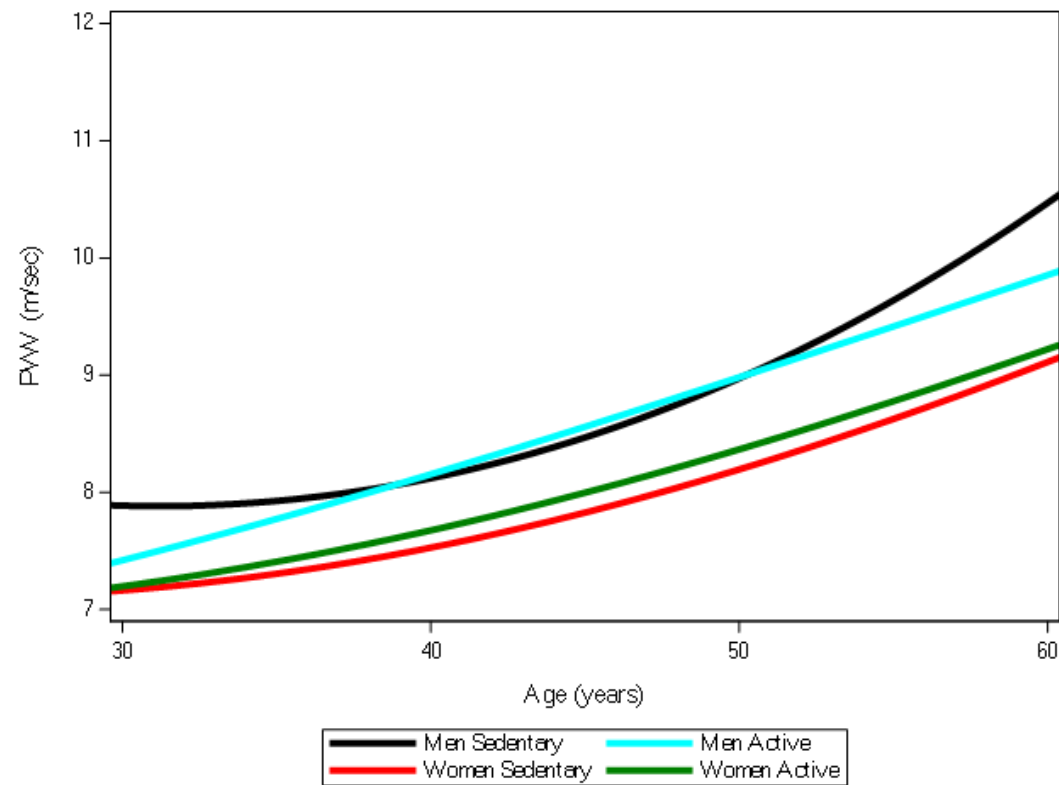

## Panel B

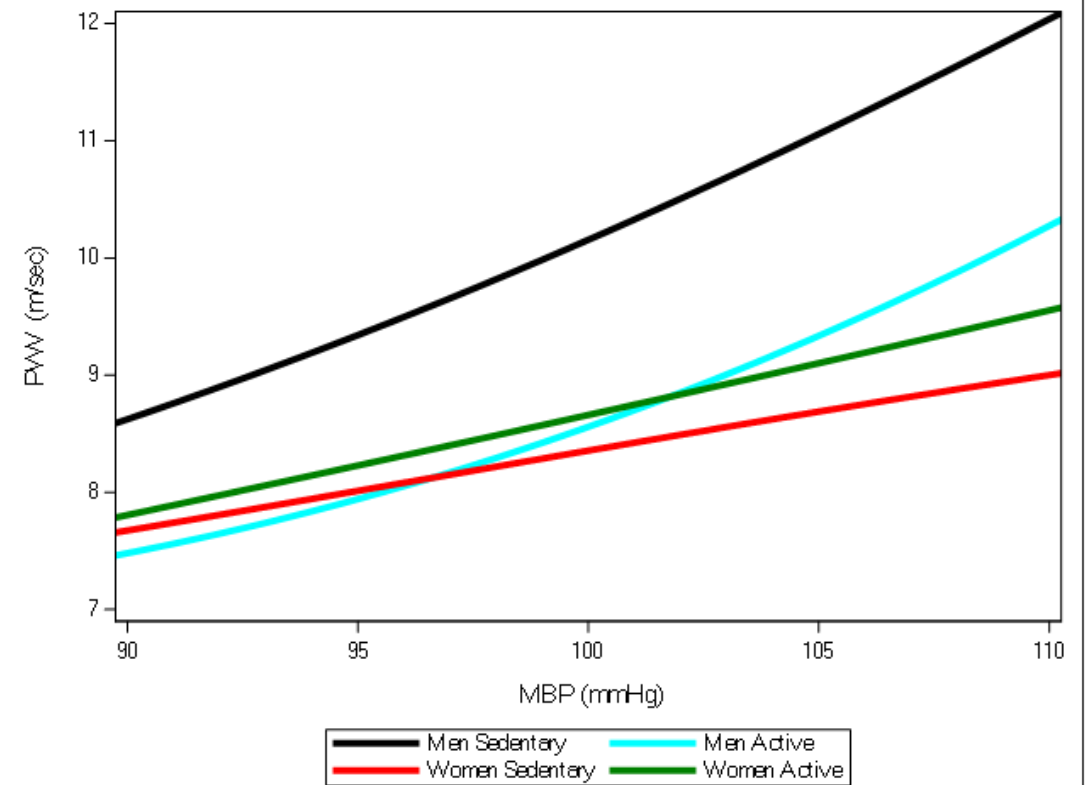

Supplement: Supplementary file 2 [file DataSheet1.pdf]
